# Can Recent Global Changes Explain the Dramatic Range Contraction of an Endangered Semi-Aquatic Mammal Species in the French Pyrenees?

**DOI:** 10.1371/journal.pone.0159941

**Published:** 2016-07-28

**Authors:** Anaïs Charbonnel, Pascal Laffaille, Marjorie Biffi, Frédéric Blanc, Anthony Maire, Mélanie Némoz, José Miguel Sanchez-Perez, Sabine Sauvage, Laëtitia Buisson

**Affiliations:** 1 Conservatoire d’Espaces Naturels Midi-Pyrénées, Toulouse, France; 2 CNRS, UMR 5245, EcoLab (Laboratoire Ecologie Fonctionnelle et Environnement), Toulouse, France; 3 Université de Toulouse, INP, UPS, EcoLab (Laboratoire Ecologie Fonctionnelle et Environnement), Université Paul Sabatier, Toulouse, France; 4 Université de Toulouse, INP, UPS, EcoLab (Laboratoire Ecologie Fonctionnelle et Environnement), ENSAT, Castanet-Tolosan, France; Ecologie, Systématique & Evolution, FRANCE

## Abstract

Species distribution models (SDMs) are the main tool to predict global change impacts on species ranges. Climate change alone is frequently considered, but in freshwater ecosystems, hydrology is a key driver of the ecology of aquatic species. At large scale, hydrology is however rarely accounted for, owing to the lack of detailed stream flow data. In this study, we developed an integrated modelling approach to simulate stream flow using the hydrological Soil and Water Assessment Tool (SWAT). Simulated stream flow was subsequently included as an input variable in SDMs along with topographic, hydrographic, climatic and land-cover descriptors. SDMs were applied to two temporally-distinct surveys of the distribution of the endangered Pyrenean desman (*Galemys pyrenaicus*) in the French Pyrenees: a historical one conducted from 1985 to 1992 and a current one carried out between 2011 and 2013. The model calibrated on historical data was also forecasted onto the current period to assess its ability to describe the distributional change of the Pyrenean desman that has been modelled in the recent years. First, we found that hydrological and climatic variables were the ones influencing the most the distribution of this species for both periods, emphasizing the importance of taking into account hydrology when SDMs are applied to aquatic species. Secondly, our results highlighted a strong range contraction of the Pyrenean desman in the French Pyrenees over the last 25 years. Given that this range contraction was under-estimated when the historical model was forecasted onto current conditions, this finding suggests that other drivers may be interacting with climate, hydrology and land-use changes. Our results imply major concerns for the conservation of this endemic semi-aquatic mammal since changes in climate and hydrology are expected to become more intense in the future.

## Introduction

Forecasting global change impacts on biodiversity is a crucial and urgent challenge [1]. The use of modelling tools such as Species Distribution Models (SDMs) may help to understand how species shift their distribution over time and to predict the impacts of future climate change on these range shifts [2]. This statistical modelling approach has its foundations in ecological niche theory [3–4]. The fundamental niche represents the environmental conditions where a species can persist while the realized niche is the part of the fundamental niche that is occupied. This latter niche accounts for biotic interactions (e.g. competition, predation, parasitism), population dynamics, natural or human-induced disturbances, as well as natural and artificial barriers to dispersal. Given that only abiotic variables are generally used in practice due to data availability [5], the application of SDMs assumes the equilibrium between the species’ distribution and its environment (i.e. the species occupies all environmentally suitable locations) to capture the fundamental niche as closely as possible [6].

In addition, although SDMs are frequently used to forecast future species distribution, good SDM performances for the time period on which the SDM is calibrated in do not guarantee that the changes in species distribution are correctly estimated [7]. To assess such uncertainty, some studies have projected distributional shifts through time using SDMs calibrated on historical data to forecast current-day distributions, and comparing the observations and the predictions [7–12]. Nevertheless, these studies remain limited to few taxa (e.g. plants, butterflies, birds) suggesting that the accuracy of SDMs to correctly track species’ range changes is still insufficiently understood.

The scarcity of studies is particularly marked for freshwater organisms whereas ecosystems hosting these species are amongst the most threatened throughout the world [13]. Among the imperiled freshwater fauna, a majority of studies has focused on fish to understand the effects of climate change on biodiversity [14–18], probably due to their expected sensitivity to temperature modifications. Yet, broadening our understanding of the effects of global change to other freshwater taxa is required to assess more comprehensively the vulnerability of freshwater ecosystems. For instance, few studies have been conducted on semi-aquatic mammals (but see in [19–24]), in spite of the numerous endangered species [25]. Among them, the Pyrenean desman (*Galemys pyrenaicus*) is a semi-aquatic mammal endemic to the Pyrenees (France, Spain and Andorra) and to the northern mountains of the Iberian Peninsula. This small mammal is strongly dependent on aquatic habitats owing to its feeding regime mainly made up of aquatic larvae of invertebrates [26]. It makes it increasingly threatened by human-induced modifications of its aquatic environment (e.g. hydro-electricity, water quality, degradation of river banks). In addition, both the fragmentation of its narrow range and recent observations suggesting a decline in its populations [27] justify the international priority given to the Pyrenean desman for conservation and management efforts [27–29]. Being restricted to aquatic habitats in mountainous regions, the Pyrenean desman has limited dispersal abilities and its high sensitivity to climatic conditions has been recently shown [20, 30]. This overall unfavorable context may thus make this species highly vulnerable to the effects of global change.

To date, SDMs have been widely used to assess climate change impacts on biodiversity since climate is acknowledged as the major factor influencing species distribution at large scale [11, 19, 31–33]. However, non-climatic components of global change (e.g. habitat fragmentation or degradation, land-use changes, urbanization) [34] can also contribute to species range shifts [35–36]. The inclusion of other environmental factors may thus improve the estimation of species distribution patterns at both local [37] and broader [10, 38] spatial scales. In freshwater environments, few studies have considered land use [39, 40] and/or hydrological [15, 41–42] changes in addition to climate change. The great importance of these factors for aquatic species distribution is, however, well known [43, 44]. The lack of studies including hydrological variables in SDMs is likely due to the non-availability of detailed data for the whole stream network at large spatial scale [45]. A solution to counterbalance the lack of accurate hydrological data is to simulate spatially and temporally flow variables using hydrological models (e.g. the Soil and Water Assessment Tool, SWAT; the PREcipitation-Runoff-EVApotranspiration HRU model, PREVAH; the Regional Hydro-Ecologic Simulation System, RHESSys), before using flow-derived metrics as input variables in SDMs. Despite promising results, few studies have applied this kind of integrated modelling approach [42, 44–46]. Moreover, the challenge of studying the effects of global change on the distribution of aquatic species is intensified by the dendritic configuration of rivers which constrains species dispersal and consequently restricts their capacities to spatially track suitable environmental conditions [47]. Indeed, SDMs in freshwater environments are frequently applied without differentiating terrestrial and aquatic realms [48].

In this context, our goal was to address the above-mentioned issues in SDMs for freshwater organisms while focusing on the Pyrenean desman, a threatened species. Our main questions were: (1) Are hydrological variables good indicators to explain the distribution of a semi-aquatic species at a broad scale, but at a finer resolution comparatively to other environmental factors (e.g. climate, land-use)? (2) Has the distribution of the Pyrenean desman shifted over the last decades? (3) Are SDMs accounting for climate, land-use and hydrological changes able to accurately predict the current species distribution when forecasted over a time period experiencing rapid environmental changes?

## Material and Methods

### Study area

The French Pyrenees (W1°400–E3°100, N43°080–N42°230) are a mountain range approximately 400 km, running from the Atlantic Ocean to the Mediterranean Sea in southern France, covering an area of approximatively 18 000 km^2^ with a maximum elevation of 3 298 m. Land cover across the French Pyrenees is dominated by woodland (38%; Corine Land Cover DB, map of the European environmental landscape, 2006) while agricultural areas are concentrated in the foothills (37%). Pastures are mainly located at medium elevation and bare rock dominates in areas of highest elevation. The population density (on average, 28.4 inhabitants per km^2^ in 2010; INSEE, 2010) is low compared to the average density in France (i.e. 115.4 inhabitants per km^2^). Annual rainfall, air temperature and stream flow greatly vary from west to east. Eastern Pyrenees are characterized by a drier and warmer climate associated with a greater seasonal variability of stream flow than the western Pyrenees.

The hydrographic network of the French Pyrenees is dense with about 26 812 km of streams (CARTHAGE DB, 2011). In this study, we considered stream reaches of approximatively 1 km-long as the unit of analysis, which is more appropriate for aquatic organisms than catchments or grids that include terrestrial environments [49]. The hydrographic network was divided into 1 km-long sections (hereafter called ‘sections’) for the computation of environmental covariates and statistical analyses.

### Field sampling

Each sampling site corresponded to a 500 m-long riverbed transect, which approximately matches the mean home range of the Pyrenean desman [50].

Two temporally-distinct sampling surveys of the Pyrenean desman were conducted. For the historical survey, the study area was divided into 232 grid cells of 10 x 14 km [51]. Between two and six sampling sites were surveyed in each cell to obtain an even coverage of the study area. In total, 637 sites where the presence or absence of the Pyrenean desman was determined were sampled between 1985 and 1992 (S1a Fig).

The current presence-absence data for the Pyrenean desman were collected between 2011 and 2013 [27]. A total of 1222 sampling sites were surveyed (S1b Fig). Their spatial location was derived from two sampling designs: (1) a resampling of historical sampling sites (in total, 514 sites were common to historical and current periods; 123 historical sites were not resampled due to fieldwork constraints such as difficult access or dried streams), and (2) a spatially balanced sampling design (i.e. Generalized Random Tessellation Stratified sampling) which is known to be suitable to study aquatic systems [52].

Most sampling sites for both the historical and current surveys were located in areas where no specific permissions were required (i.e. state-owned streams). Those located on private land were accessed only after the owner gave permission to conduct sampling. All sampling sites did not involve endangered or protected species. The potential disturbance induced by wading the stream section could not be avoided but remained as minimal as possible.

Given the cryptic behaviour of this species, indirect signs (i.e. faeces) were searched along these sampling sites which were waded by skilled observers meticulously inspecting each emergent item (i.e. rock, tree root or branch) in the riverbed. Samplings were not conducted during or after a period of fluctuating water levels or heavy rainfall to maximize the detectability of faeces [44, 51]. For each period, sampling was conducted following a similar methodology and always under good environmental conditions which promised little bias in desman faeces detectability.

### Environmental variables

We first obtained a large set of environmental variables describing hydrology, hydrography, climate, land use and human disturbance that are expected to influence the distribution of the Pyrenean desman. These variables were selected *a priori* based upon our current limited knowledge of the biology and ecological requirements of the Pyrenean desman [27], but also those of other semi-aquatic mammals (e.g. aquatic shrews) or aquatic organisms occupying similar habitats (e.g. stream fishes, water birds). We then computed pairwise Spearman correlation coefficients between variables and only variables that were not highly correlated (i.e. |rho|≤0.65) were retained, resulting in a final set of 11 environmental variables (S1 Table). All selected variables were log-transformed and normalized.

#### Climatic variables

Atmospheric mean annual temperature (TEM) and mean annual rainfall (RAI) were calculated on two 10-year periods (1976–1985 and 2002–2011 for the historical and current periods, respectively). They were generated at an 8-km spatial resolution by a statistical downscaling methodology, with the Meteo-France SAFRAN mesoscale meteorological analysis [53–54]. Mean and variability over several years are classically used in climatology to reduce the influence of years with extreme events (e.g. heatwaves) and to obtain more accurate representation [55]. The values of TEM and RAI of the 8-km cell in which each 1-km long stream section belonged to were assigned to this stream section. For stream sections crossing several 8-km cells, a weighted average accounting for the relative length in each cell was calculated.

#### Land-use variables

The 1990 and 2006 versions of the Corine Land Cover database were used to calculate the historical and current land-use variables, respectively. Land-use variables described the proportion of forest (FOR), urban areas (URB), agricultural land (AGR) and open space with little or no vegetation (NAT). The percentage of each variable was calculated in a 100 m-buffer around each river section as recommended by the Riparian Forest Buffer Initiative (http://dnr2.maryland.gov/forests/Pages/programapps/ripfbi.aspx).

#### Hydrological variables

We used one of the most applied hydrological models worldwide, SWAT [56], to simulate the mean monthly stream flow (FLO) in the whole stream network of the French Pyrenees. Based on spatial information (i.e. topography, climate, soil and land-use), SWAT simulates the hydrological cycle both in space and time, (see [56] for details). SWAT requires several input datasets through the ArcSWAT interface in ArcGIS 10.0 [57]. First, the French Pyrenees were divided into 29 915 sub-basins (mean area = 79.20 ha ± 72.84) with a discretization scale of 40 ha by using a 25 m resolution Digital Elevation Model (ALTI DB–IGN, 2011). To characterize soil and land-use conditions, a 1 km^2^ resolution Digital Soil Map of the World (FAO, 2007), and a 500 m resolution land cover map (Corine Land Cover; see above) were also included, respectively. Climatic variables used to calibrate SWAT models consisted of daily rainfall, maximum and minimum air temperature, solar radiation, wind speed and relative humidity [54] from SAFRAN mesoscale meteorological analysis based on measurements (8 x 8 km). We modified snow parameters to calibrate SWAT and improve simulations (S2 Table). Simulated stream flows were finally obtained for each sub-basin at monthly time resolution for the two 10-year periods (i.e. 1976–1985 and 2002–2011 for the historical and current periods, respectively). The flow value assigned to each 1-km river section corresponds to the one of the sub-basin in which it is included. It means that several 1-km stream sections belonging to the same sub-basin were assigned the same stream flow value. In the rare cases where a 1-km stream section crossed several sub-basins, the assigned stream flow value was the one of the sub-basin containing the longest portion of the 1-km stream section. In addition, measured monthly stream flow data were available at 24 and 30 downstream gauging stations for the historical and current periods, respectively (23 stations common to both periods; see S2 Fig), and were used to calibrate and validate SWAT flow simulations. SWAT model was calibrated on the first five years of each 10-year period and validated on the last five years. The accuracy of SWAT simulations was assessed using three different metrics: the Spearman correlation coefficient (rho), the coefficient of determination (R^2^) and the Nash–Sutcliffe Efficiency (NSE), each of them being calculated between the measured and simulated stream flow [58–59]. According to Moriasi et al. [60], SWAT flow simulations were accurate, as indicated by the evaluation statistics averaged across the gauging stations used for the validation step of each time period (historical period: rho = 0.79 ± 0.21; R^2^ = 0.66 ± 0.27; NSE = 0.24 ± 0.93; current period: rho = 0.83 ± 0.18; R^2^ = 0.67 ± 0.24; NSE = 0.36 ± 0.56). Mean evaluation statistics were also satisfying at the time period used for the calibration step (historical period: rho = 0.81 ± 0.14; R^2^ = 0.65 ± 0.22; NSE = 0.25 ± 0.69; current period: rho = 0.81 ± 0.16; R^2^ = 0.68 ± 0.23; NSE = 0.51 ± 0.37).

#### Hydrographic variables

The mean slope of the section (SLO) and the number of tributaries in the focal section and in its adjacent upstream and downstream sections (TRI) were calculated using a 25 m resolution Digital Elevation Model (see above) and the French national hydrographical database (CARTHAGE DB, 2011). The slope is a good surrogate for elevation and water energy that may both influence the distribution and dispersal of the Pyrenean desman [44]. The tributaries may act as refuges in case of disturbance in the main channel (e.g. variation in hydrology, water pollution). Thus, the number of tributaries surrounding the stream section reflects the density of potentially suitable habitats for the Pyrenean desman. These two variables were assumed to remain unchanged (i.e. static) between the two periods.

#### Human disturbance variables

The density of obstacles to water flow (e.g. dams, weirs) upstream of the focal river section (OBS; ROE ^®^ DB, 2013) and the human population density (POP; GEOFLA ^®^ DB, 2014) in a 100 m-buffer around each river section were calculated to describe river fragmentation and the degree of human disturbance. These two variables were also assumed to remain unchanged (i.e. static) between the two periods.

#### Comparison between historical and current environmental conditions

We compared the similarity of environmental conditions between both periods for (1) sampling sites and (2) the whole study area using the Istat function of the SDMTools package of the R environment [61] which computes the I similarity statistic [62] for quantifying niche overlap. This statistic ranges from a value of 0, where two distributions have no overlap, to 1 where two distributions are identical.

### Species distribution modelling

#### Modelling framework

**Fig 1:**
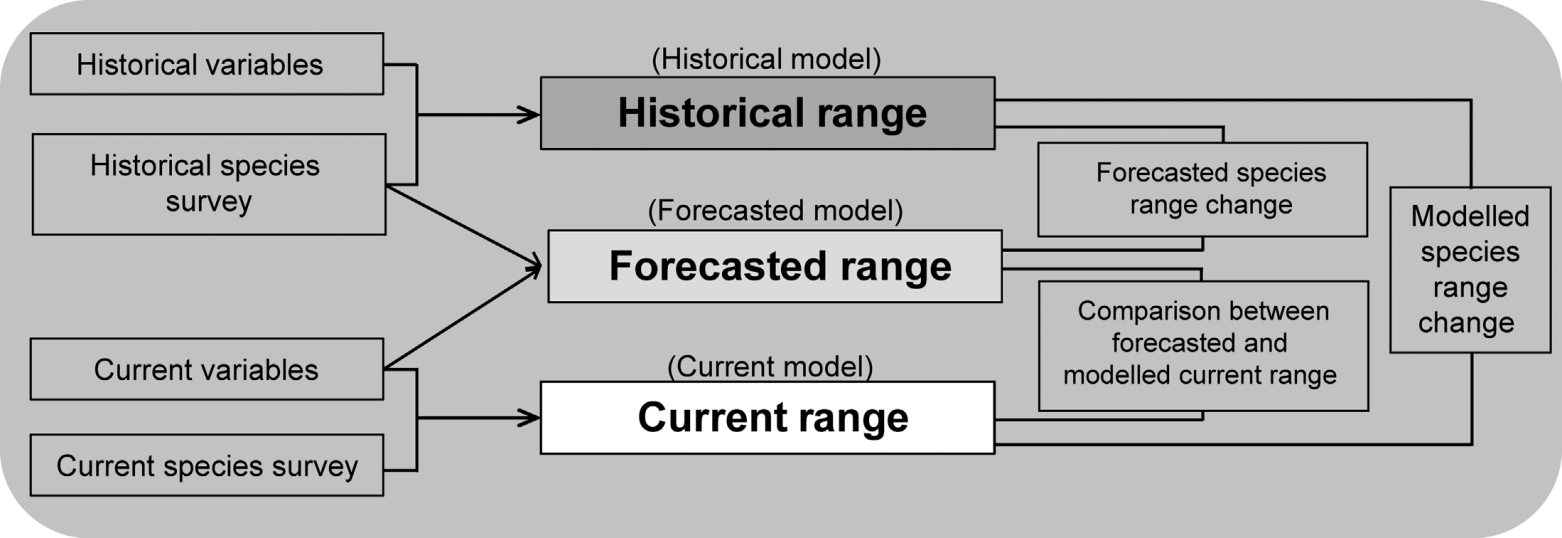
Methodology used to build the historical, forecasted and current distribution models for the Pyrenean desman in the French Pyrenees. Modified from [9, 63].

Following Kharouba et al. [9] and Grenouillet et al. [63], the modelling procedure was divided into three steps (see Fig 1). First, we used the historical species presence-absence and historical environmental variables to build the historical model and predict the historical range of the Pyrenean desman. Secondly, the historical model was applied to the current values of the environmental variables (i.e. the forecasted model) to forecast the current suitable habitats for the species while assuming ecological niche conservatism [64–65]. Finally, we used the current species’ presence-absence and environmental variables to build the current model and predict the current range of the Pyrenean desman.

For both historical and current periods, presence-absence data of the Pyrenean desman were related to the 11 environmental variables (S1 Table) through an ensemble modelling approach [66]. The package “BIOMOD” [67] of the R software [61] was used to run six different algorithms: Generalised Linear Models (GLM), Generalised Additive Models (GAM), Generalised Boosting Models (GBM), Artificial Neural Networks (ANN), Multivariate Adaptive Regression Splines (MARS) and Random Forests (RF). We used default values of parametrization [67]. For each period, SDMs were built using a random subset of data containing 80% of the sites (i.e. calibration dataset) and the remaining 20% were used to evaluate the predictive performance of the models (i.e. validation dataset). This process was repeated 30 times using different calibration and validation datasets at each iteration. The predictions were averaged (i.e. simple average) across the 30 iterations and the performance of the ensemble model (i.e. the average of the six predictions, one by algorithm) was evaluated on the validation dataset using a threshold independent measure (i.e. the area under the receiver operating curve; AUC) [68]. In addition, as the AUC has been reported to be misleading in some cases [69], we also computed the True Skill Statistics (TSS) to evaluate the predictive accuracy of the models.

For each of the historical, forecasted and current models, the probabilities of occurrence of the Pyrenean desman were then predicted over the entire stream network of the French Pyrenees. The mean predicted occurrence probability for each river section was calculated across the 180 model outputs (i.e. 6 algorithms x 30 iterations) to produce three final habitat suitability maps (i.e. one for each model). The relative importance of each environmental variable was evaluated by computing the Pearson correlation coefficient between initial model predictions and model predictions obtained by randomly permuting the variable of interest [67].

#### Comparison of the historical, forecasted and current models

First, we compared the response curves of the environmental variables influencing the distribution of the Pyrenean desman the most for the historical and the current periods. Changes in response curves may inform about changes in the realized ecological niche, which could result in range shifts. Response curves were obtained by using the response.plot function of BIOMOD [70]. For building the predicted response curves, n-1 variables are set constant to a fixed value and only the remaining one is varying across its whole range. This function enables to plot the response curves of a model independently of the algorithm used for building the model. It therefore permits a direct comparison of predicted responses from the different statistical approaches on the same data. Then, for each variable, response curves of the six different SDMs were averaged. We also tested the accuracy of the forecasted model to predict the current distribution of the species by calculating the AUC between the forecasted range and the entire current survey of the species (Fig 1).

Secondly, we compared the forecasted and the current habitat suitability maps by calculating the relative change between forecasted and current occurrence probabilities. A positive value indicates an overestimation of the forecasted current occurrence probability compared to the modelled current range, while a negative value indicates that the forecasted model underestimates habitat suitability compared to the current observations. A value of zero indicates perfect agreement between both maps.

Thirdly, the forecasted range change was similarly estimated by comparing historical and forecasted habitat suitability maps. The relative change between historical and forecasted occurrence probabilities was calculated.

Finally, the modelled range change was also estimated by comparing historical and current habitat suitability maps. It was computed through the relative change between historical and current occurrence probabilities.

## Results

### Comparison of historical and current datasets

During the historical survey, faeces of the Pyrenean desman were detected in 519 out of the 637 sampling sites, resulting in a species prevalence of 81% while they were found in only 46% of the sampling sites (i.e. 557 out of 1222 sites) during the current survey (S1 Fig). A direct comparison of the presence-absence at the 514 sites sampled in both periods indicated that the Pyrenean desman has not been detected in the current survey in 29% of the sites where it was present in the past.

The environmental conditions at sampling sites encompassed the same range of environmental conditions for both time periods (S3 Fig) which are representative of the conditions encountered in the entire French Pyrenees. This suggests no potential extrapolation outside the variables range when forecasting the distribution of the Pyrenean desman in the current period but also that the outputs of historical and current models can be compared. Additionally, there was a strong similarity between both periods regarding environmental conditions available at sampling sites and in the whole study area (I similarity statistic > 0.95 for all environmental variables).

Over the last three decades, an increase in air temperature has been measured for all river sections with a mean increase of 0.5°C across the French Pyrenees (Table 1). The greatest warming has occurred at the highest elevations (S4 Fig). A consistent decrease in annual rainfall has also been measured over the study area with an average difference of 166.78 mm (Table 1). The strongest reductions in rainfall were observed in lowland areas of the central and western Pyrenees (S4 Fig). On average across the study area, land-use changes between the historical and current periods have been very small (average changes below 1%; Table 1; S4 Fig).

**Table 1:** Measured changes in the environmental variables between historical and current periods across the French Pyrenees.

Variable	Historical	Current	Range change (%)	Mean change ± sd (%)
**TEM (°C)**	**10.81**	**11.30**	**+2.98 to +34.27**	**+5.13 (± 2.71)**
**RAI (mm)**	**1216.60**	**1049.82**	**-18.31 to -4.90**	**-13.52 (±2.42)**
**URB (%)**	**2.44**	**2.77**	**-46.41 to +99.97**	**+0.32 (±3.35)**
**AGR (%)**	**37.37**	**37.03**	**-100 to +95.71**	**-0.33 (±6.98)**
**FOR (%)**	**38.04**	**38.13**	**-100 to +99.99**	**+0.09 (±6.10)**
**NAT (%)**	**21.51**	**21.32**	**-95.74 to +99.98**	**-0.20 (±7.64)**
**FLO (m**^**3**^**/s)**	**1.69**	**1.47**	**-48.55 to 119.11**	**-11.72 (±18.34)**

For climatic (TEM, RAI) and stream flow (FLO) variables, the historical and current periods correspond to 1976–1985 and 2002–2011, respectively whereas they correspond to 1990 and 2006 for land-use variables. The values given in the second and the third columns show the average conditions across the study area.

Simulated mean monthly flow ranged spatially from 0.01 to 140.38 m^3^/s (mean = 1.69 m^3^/s) and from 0.01 to 116.29 m^3^/s (mean = 1.47 m^3^/s) for the historical and the current periods, respectively (Table 1; S4 Fig). Overall, predicted stream flow was lower for the current period than for the historical one except for some river sections located in the eastern Pyrenees whose stream flow was predicted to increase (maximum increase = +119.11%).

### Historical predicted range of the Pyrenean desman

**Fig 2:**
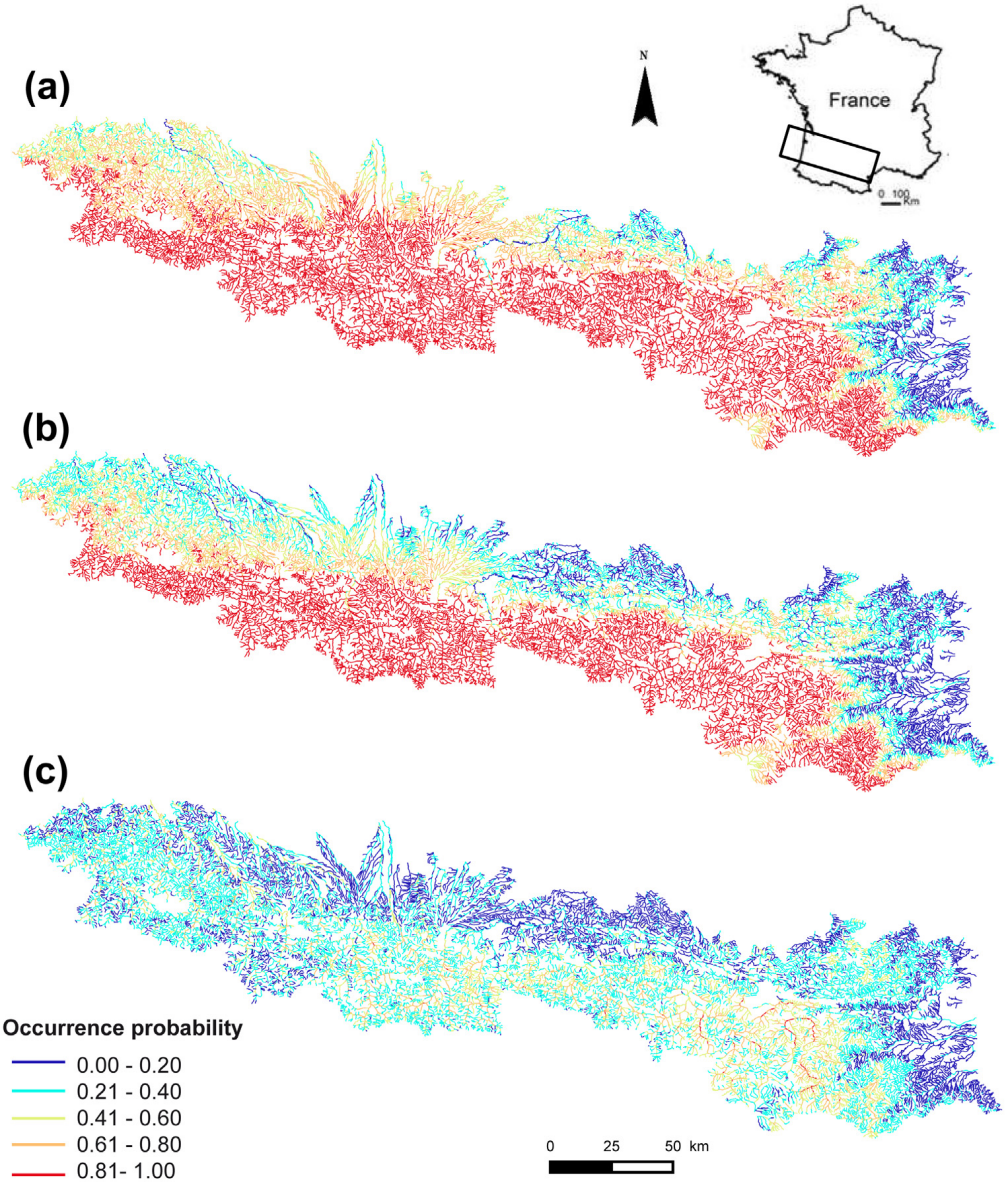
Occurrence probabilities of the Pyrenean desman predicted across the French Pyrenean stream network. (a) historical, (b) forecasted and (c) current models.

**Fig 3:**
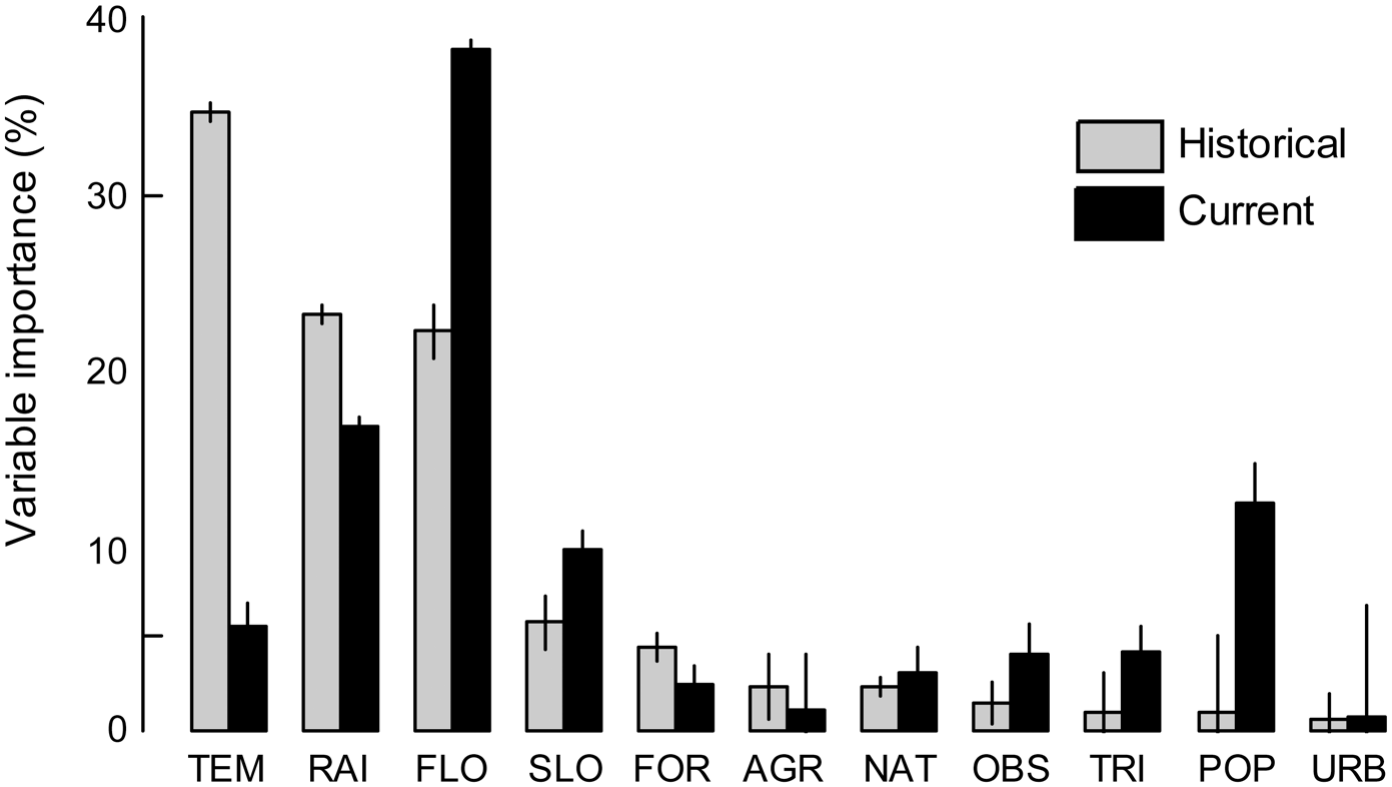
Relative contribution (%) of each environmental variable in the models for the two periods. The historical period is depicted in grey while the current period is shown in white. Barplots indicate the mean importance (± standard error) across the six modelling methods. The environmental variables are sorted by decreasing importance for the current period.

**Fig 4:**
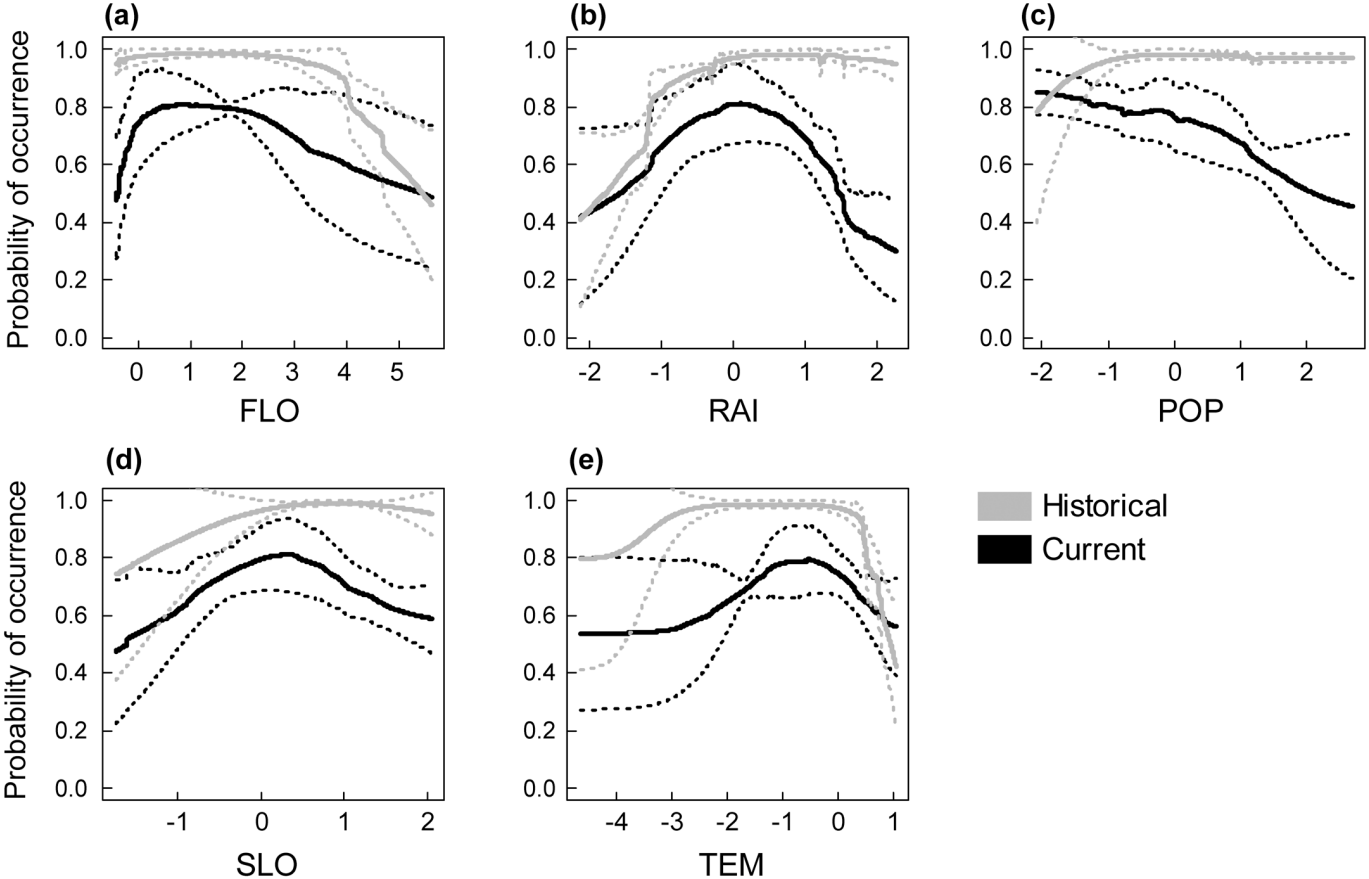
Response curves of the most relevant environmental variables influencing the distribution of the Pyrenean desman. Response curves for the historical predictions are depicted in grey while those for the current prediction are shown in black. Solid lines indicate the mean response across the six statistical models while dotted lines represent the 95% confidence intervals. All non-categorical covariates were log-transformed and normalized.

The predictive performance of the historical model was high (AUC = 0.83 ± 0.03 and TSS = 0.57 ± 0.04 across the six modelling techniques; mean ± SD). The predicted occurrence probabilities were quite high too (mean ± SD of 0.70 ± 0.27), with 20 373 km of stream (i.e. 76%) having occurrence probabilities higher than 0.5 (Fig 2a). Climatic and hydrological variables contributed the most to the Pyrenean desman’s distribution prediction, with relative contribution higher than 20% for TEM, RAI and FLO (Fig 3). Land-use and human disturbance variables had a small influence (i.e. less than 5%). The response curves highlighted a positive relationship between the mean annual rainfall and the Pyrenean desman’s probability of occurrence (Fig 4b). In contrast, a negative influence of the mean annual temperature was found with a strong decline of occurrence probabilities for the warmest stream sections (Fig 4e). Very high occurrence probabilities were predicted in stream sections showing low to medium values of stream flow, however these decreased with increasing flow rate (Fig 4a).

### Current predicted range of the Pyrenean desman

The predictive performance of the current model was fair with a mean AUC of 0.70 (± 0.02) and a mean TSS of 0.35 (± 0.03). The predicted occurrence probabilities were quite low (0.32 ± 0.17) with only 4 224 km of stream (i.e. 16%) having occurrence probabilities higher than 0.5 (Fig 2c). This suggests a current low suitability of many habitats of the French Pyrenees for this species, especially for the most downstream areas and for the easternmost and westernmost parts of the Pyrenees. Similarly to the historical period, the hydrological and climatic variables influenced the distribution of the Pyrenean desman the most with relative importance above 15% for FLO and RAI (Fig 3). The mean annual temperature had a much lower influence in the current model than in the historical model (5.80 ± 5.47%) while the relative importance of stream flow was higher for the current period than the historical one (38.17 ± 15.39%). The human population density and the slope of the stream section also contributed in explaining the current species distribution (i.e. between 10 and 15% for each variable) while the land-use variables were the least influential of all. A bell-shaped response of the occurrence probability was highlighted for the mean annual temperature (Fig 4e), the mean annual rainfall (Fig 4b) and the section slope (Fig 4d). Last, the population density negatively influenced the current occurrence probability of the Pyrenean desman (Fig 4c).

### Forecasted species range change (historical vs. forecasted models)

**Fig 5:**
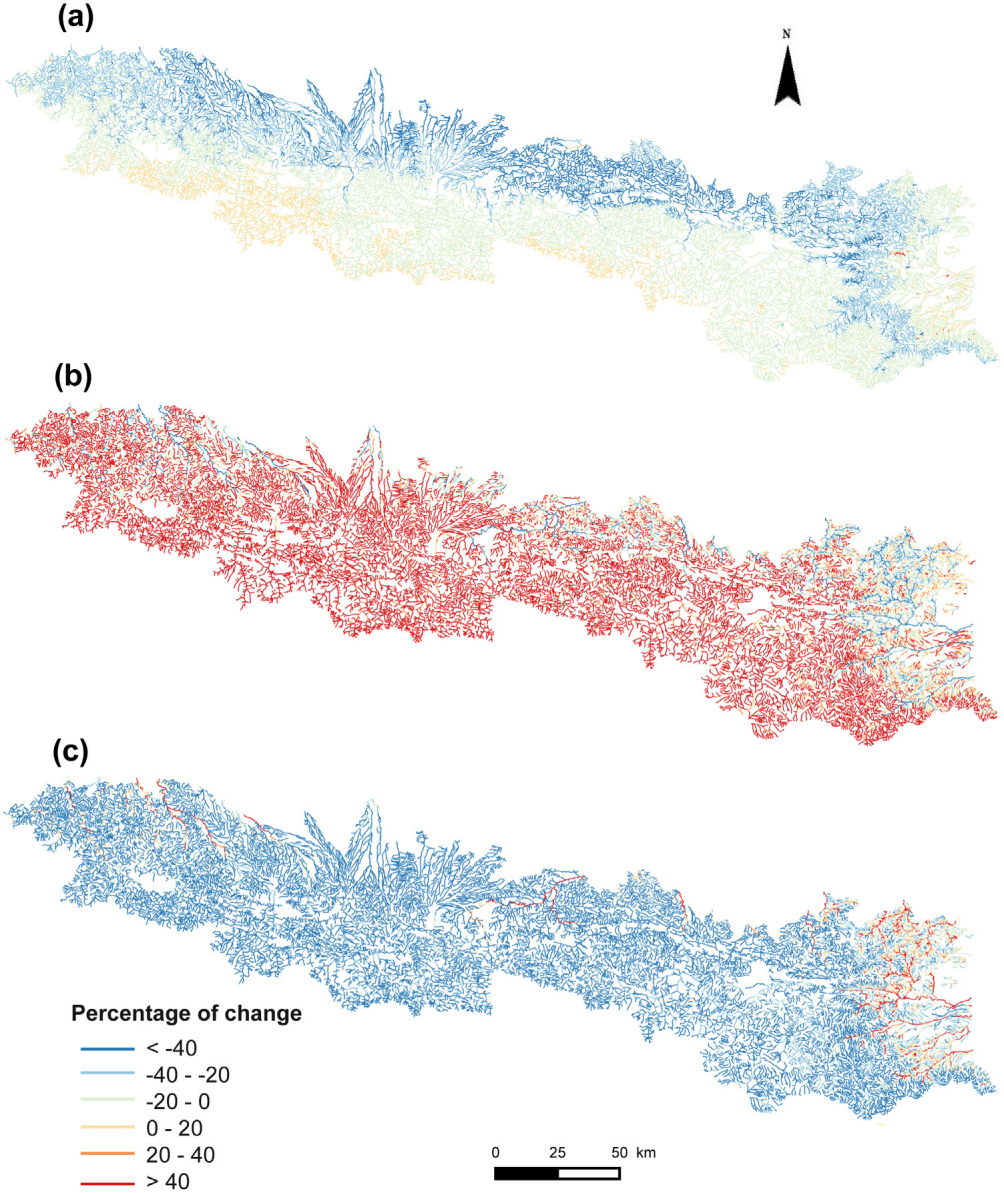
Percentage of change in predicted occurrence probabilities of the Pyrenean desman. Percentage of change between (a) the historical and forecasted models (i.e. expected range change), (b) the current and forecasted models, and (c) the historical and current models (i.e. modelled range change).

**Fig 6:**
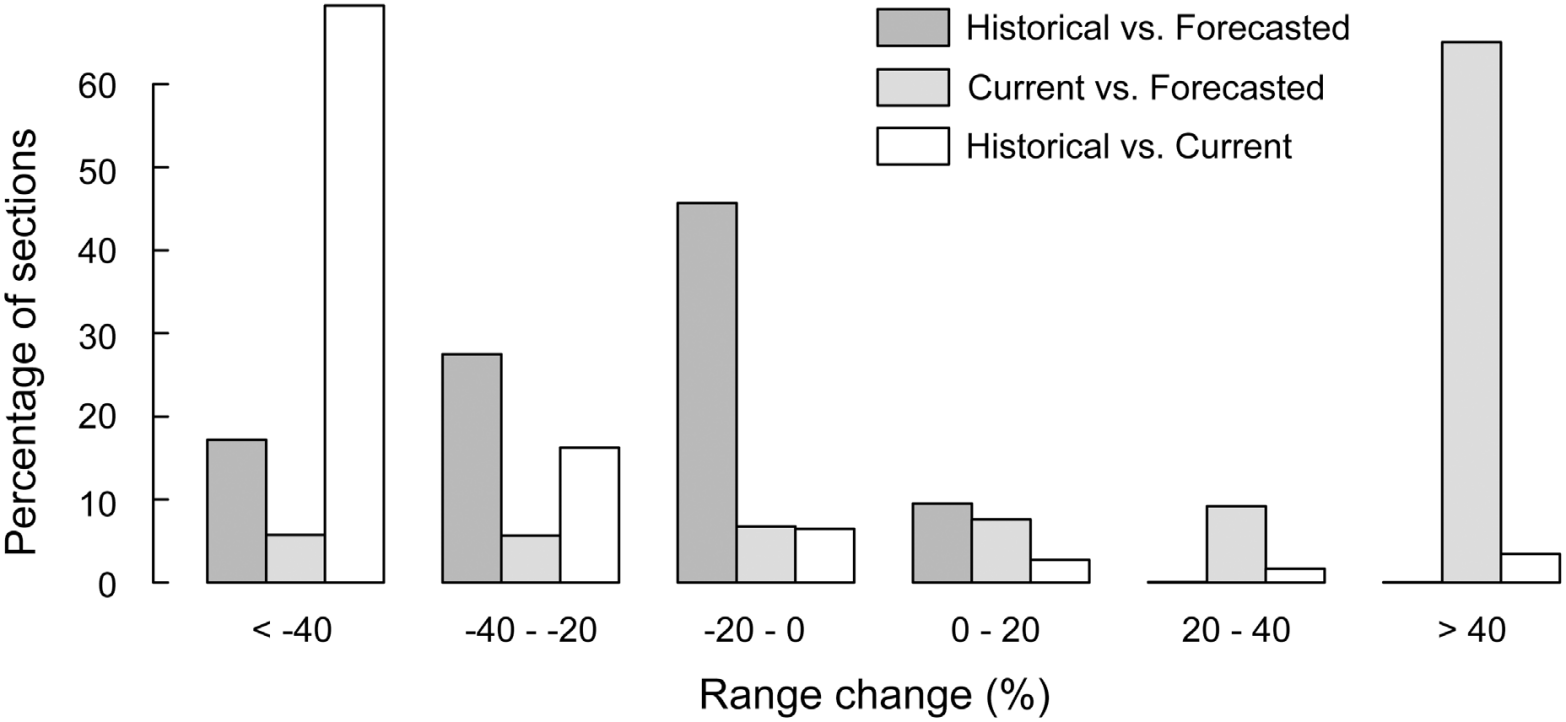
Distribution of occurrence probabilities changes. Changes between the historical and forecasted models (i.e. expected range change) are shown in dark grey, the ones between the forecasted and current models in light grey, and the ones between historical and current models (i.e. modelled range change) in white.

The forecasted model was expected to predict the current suitable habitat for the Pyrenean desman while accounting for climatic, hydrological and land-use changes that have occurred between the historical and current time periods. Overall, the recent climatic, hydrological and land-use changes have caused an average decrease of the habitat suitability of about -20% for this species in the French Pyrenees (i.e. lower occurrence probabilities; mean ± SD = 0.59 ± 0.31), with 24 252 km of streams out of the 26 812 km of the study area experiencing a decrease in probability of occurrence (Figs 2b and 5a). More specifically, the suitability of habitats seems to have shifted towards higher elevations but has greatly decreased in the most downstream areas (i.e. loss higher than 40%) (Fig 5a). However, most river sections (46%) were predicted to only have a slightly lower environmental suitability (i.e. loss between 0 and 20%; Fig 6).

### Comparison between forecasted and modelled current range (forecasted vs current models)

The predictive performance of the forecasted model, evaluated with the current Pyrenean desman presence-absence, was quite low (AUC = 0.56 and TSS = 0.11), suggesting that the forecasted model was poorly accurate in predicting the current species range. More specifically, the forecasted model was found to greatly overestimate the habitat suitability relative to the current predicted distribution of the Pyrenean desman in most stream sections, except for some downstream areas and for the eastern part of the French Pyrenees where the forecasted model tended to underestimate the habitat suitability (Fig 5b).

### Modelled species range change (historical vs current models)

When comparing the predictions of the historical and current models, we found that 69% of the river sections showed decreasing habitat suitability by more than 40% over the study period (mean ± SD = -45.9 ± 39%; Figs 5c and 6). Less than 8% of the stream sections representing about 2100 km of streams experienced an improvement in their predicted habitat suitability (Fig 6). These sections were located in the easternmost parts of the Pyrenees and in the downstream areas of the main rivers. These results suggest an important range contraction of the Pyrenean desman in the French Pyrenees for the last 25 years.

## Discussion

Our study has involved (1) two robust surveys at different periods of the endangered Pyrenean desman distribution that covered a wide range of environmental conditions at a fine stream reach resolution across a large geographical extent, (2) an integrated modelling approach that combines the outputs of a hydrological model with SDMs based on high spatial resolution, and (3) species occurrence predictions for historical and current time periods.

We emphasized that the current geographical range of the Pyrenean desman in the French Pyrenees has dramatically shrunk compared to the historical one, over a short time lapse (i.e. about 25 years), particularly in the western Pyrenees. More than two thirds of the stream network of the French Pyrenees has experienced a decrease in species occurrence probability by more than 40%. Our results confirm the documented decline of the Pyrenean desman in the Central System in Spain between 1838 and 2011 [29]. Alarmingly, the current predicted range size is much smaller than the one forecasted from the historical model. It means that the forecasted model tends to predict suitable habitat for the Pyrenean desman that are in fact currently unoccupied. A similar trend has been reported by Guida et al. [12] for plant species in the Mojave Desert in Nevada (USA), which showed that habitat suitability was overestimated for all the species sensitive to precipitations. In contrast, Kharouba et al. [9] found that the current habitat suitability of butterflies in Canada tended to be higher than those predicted from an historical model forecasted onto current conditions. Other studies have highlighted that some species do not shift their distribution fast enough to follow their climatic niche [71–73], and consequently currently occupies sub-optimal areas. Our results also join those of Comte et al. [17] who emphasized that current rates of change of fish species distribution in the world are of greater magnitude that those projected when considering solely future climate change.

The range contraction of the Pyrenean desman seems to be much faster than the decrease in habitat suitability due to climate, hydrological and land use changes that have occurred for the last three decades. These findings thus suggest that some other abiotic and/or biotic factors, which were not included in the models while likely influencing the distribution of the Pyrenean desman, have rapidly changed over time explaining the considerable range contraction of the species. Among these factors, habitat fragmentation, water and substrate pollution, invasive species, local habitat degradation (e.g. shelters, riverbanks) and riparian vegetation changes are also considered to be critical for aquatic communities [74–76]. In particular, biotic interactions are generally not included in SDMs [77] although their effects on species distribution may sometimes be more important than those of climate [78], thus potentially improving SDMs performance [79–80]. Indeed, a species may be absent from an area or constrained to use a sub-optimal habitat, for instance owing to the presence of a competitor or a predator, or to the absence of a mutualist or a prey species [81]. In the present study, the strong range contraction of the Pyrenean desman in recent years could be related to the rapid expansion of the American mink *Neovison vison* in the French Pyrenees [82], which is an alien invasive species known to prey on the Pyrenean desman [83]. This recent range contraction could also be explained by potential changes in the composition of macro-invertebrate communities (i.e. the main prey of the Pyrenean desman) [51] induced by recent climate change. For instance, a study [84] reported that a period of rising temperatures coupled with low rainfall and river flows in New South Wales (Australia) resulted in a range decline of the stream macro-invertebrate families favoring cooler waters and faster flows. Domisch et al. [32] have also forecasted that more than half of the European stream macro-invertebrates could lack of climatically suitable habitats and may thus shift their distribution in the future, especially cold-adapted species. Given the recent observed warming (+0.5°C) and decrease in rainfall (-13%) and stream flow (-12%) in the French Pyrenees, such response of macro-invertebrate communities could therefore have worsened the contraction of the Pyrenean desman’s range.

Among the environmental factors included in the model, we found a strong influence of hydrological parameters on the distribution of the Pyrenean desman that is consistent with a previous study led at the scale of a single French Pyrenean catchment [44]. Stream flow has also been reported as an important factor for the Pyrenean desman regarding food availability (i.e. abundance and richness of invertebrates) and its floating behaviour [44, 85]. Climatic factors (i.e. temperature and precipitation) were also identified as relevant predictors of the Pyrenean desman distribution across the French Pyrenees, especially in the historical period. This finding is consistent with previous studies conducted at nation-wide extents [20, 37, 86] but at coarser resolution (e.g. 10 x 10 km pixels). In the current context of global change, climatic and hydrological variables are projected to be strongly modified in the coming decades [43, 87]. This suggests severe additional threats for the endangered Pyrenean desman. Indeed, the French Pyrenees could experience strong climate modifications in the next century, with a projected decrease in precipitation between 10.7 and 14.8%, and a warming by 2.8 to 4°C according to regional climate models [88]. Being restricted to mountainous areas, the Pyrenean desman has limited opportunities to shift towards higher elevations in response to climate changes as it is expected for organisms living lowland, suggesting that its range contraction could be hastened, as evidenced for other mountain plant and animal species [72]. For instance, Morueta-Holme et al. [20] forecasted a significant reduction of the range of the Pyrenean desman in Spain under future climate scenarios. In addition, its ability to move to other environmentally suitable river catchments or upstream areas is reduced due to the high natural (e.g. dendritic network, biogeographic barriers) and artificial (e.g. dams, weirs; about 2500 artificial barriers within the study area according to the database summarizing all the physical barriers to water flow in France, ROE) fragmentation of aquatic systems [89].

Compared to temperature, rainfall and stream flow, our results suggest that land use features have a small influence on the distribution of the Pyrenean desman at the scale of the French Pyrenees, which is in line with the work of Thuiller et al. [90] regarding more than 3 000 mammal, bird and plant species at the European scale. This finding is also consistent with the results of Filipe et al. [18] which have identified climate as the most influent predictor of the brown trout (*Salmo trutta*) distribution in Europe, but found that the inclusion of land use variables did not improve the predictive accuracy of their models. Although the direct influence of land use seems to be small for aquatic species compared to other aquatic drivers, the intensification of human activities along the rivers (e.g. intensive agriculture, industry, urbanization) increases the concentration in river pollutants which are likely to further alter the quality and the functioning of freshwater habitats and may induce the absence of some species [91]. Hence, the impacts of land use could be rather indirect than direct. In addition, the fact that land use seems to have a small influence on the Pyrenean desman distribution may also be due to the limited size (i.e. 100 m) of the buffer around each stream section instead of measuring an accumulated upstream effect. Some other studies have actually reported a more important effect when land use in the upper subcatchment is considered [45] suggesting that the impact of land use on aquatic species distribution would rather be cumulative than local.

A large part of the uncertainties when using SDMs comes from the reliability of sampling records. Indeed, potential biases may result from sampling that is not representative of the study area [92]. Consequently, predicted species distribution and species-habitat relationships could be erroneously estimated [93]. To overcome this issue, robust sampling designs (e.g. randomized, systematic, stratified), as those used in this study, are recommended [94]. The two temporally-distinct sampling surveys of the Pyrenean desman used the same methodology in detecting faeces but they differed in the number of sites visited (637 vs. 1222 sites). However, the range of environmental conditions in sampling sites was quite similar although they experienced an overall increase in temperature and decrease in rainfall and stream flow over the study period. This indicates that both the historical and current models were calibrated on comparable environmental conditions making possible to compare their outputs and thus supporting that the found contraction of the Pyrenean desman range was not due to biased sampling strategies.

In this study, we combined variables describing climate, land-use, hydrology, hydrographic and human disturbance in a single model while studies forecasting the distribution of biodiversity are often limited to climate variables (e.g. Worldclim). Depending on the spatial scale considered, the use of a wide diversity of environmental variables is known to better describe the ecological niche of species, thus improving the predictive performance of SDMs [37, 95]. As global change includes other components than climate and land-use changes [13, 35], taking into account additional information, such as hydrological changes, may provide more realistic assessments. As far as we know, our integrated modelling approach simulating stream flow with a good accuracy and then including predicted flow metrics in SDMs is however the first one conducted at such a fine resolution and large extent (i.e. the entire stream network of the French Pyrenees). To date, such an approach has rarely been applied at smaller (but see [44–46]) or broader (but see [42] for countrywide and worldwide scales) scales. In this study, accounting for stream flow enabled to identify areas unsuitable in terms of hydrological conditions for the Pyrenean desman, being yet located in areas with suitable climate, topography and land-use. Taking stream flow information into account is indeed important when SDMs are applied at a fine resolution [96], given that stream flow is one of the key drivers in the functioning of freshwater ecosystems [97–98]. By exerting a direct physical force, stream flow indeed structures the substrate composition, as well as the width and stability of the channel. In addition, flow rate also influences water physico-chemical properties (e.g. temperature, dissolved oxygen concentrations) which, in turn, regulate numerous environmental processes (e.g. sedimentation rate, concentration of nutrients and organic particles) [99]. Therefore, stream flow determines the heterogeneity of aquatic habitats and indirectly the distribution and diversity of organisms, such as macro-invertebrates [45], water birds [100] or aquatic mammals [101]. Integrated modelling approaches coupling hydrological models with SDMs have already shown promising results [44–46] but need to be explored further. For instance, since flow can be accurately simulated at fine time resolution (e.g. daily, monthly), other hydrological metrics could be derived (e.g. seasonality, velocity, depth, shear stress), including variables describing extreme events (e.g. high flow frequency, drought) [42, 75] which can exclude sensitive species and restructure food webs through the homogenization of aquatic habitats [100]. Besides, in spite of an overall good fit of stream flow simulations to the observations, we noticed that the accuracy of historical SWAT model was lower than the current one. This may result from the quality of the data used to calibrate (i.e. climate) and validate (i.e. measured stream flow at gauging stations) the SWAT model which were less reliable in the past than in the present. In parallel, it is also worth noting that stream flow had less influence on the distribution of the Pyrenean desman in the historical period than in the current period suggesting that the importance of hydrological variables is also influenced by their inherent quality. Hence, it seems crucial to first refine the outputs of hydrological models to get the best predictors before coupling them to ecological models such as SDMs.

Another important point to consider is the fact that the Pyrenean desman range is not limited to the French part of the Pyrenees. It occurs actually also in parts outside the study area (i.e. southern Pyrenees and northern mountains of the Iberian Peninsula) where the environmental conditions, especially climate and hydrology, can be different from those encountered in France (e.g. drier). Hence, the range of environmental conditions we used to model the distribution of the Pyrenean desman may be truncated. As a consequence, the habitat suitability in areas with other combinations of environmental variables than those of the study area may be underestimated.

Despite these few shortcomings, the results of this study stress that the Pyrenean desman may be severely at risk owing to climate change and other pervasive threats such as the degradation and the fragmentation of aquatic habitats. We have highlighted that the recent hydrological and climate changes only explain a small part of this observed decline, suggesting that this shift has likely been driven by other abiotic or biotic factors. Freshwater biodiversity managers should thus urgently initiate ambitious conservation plans to protect the aquatic habitats that are still suitable for the Pyrenean desman while simultaneously exploring the other factors that have possibly caused this range contraction. Accounting for the effects of future global changes on this endangered species is also essential when designing conservation actions to avoid implementing actions that are likely to be effective only for a short period of time.

## Supporting Information

S1 Fig Observed presence and absence of the Pyrenean desman for the historical and current surveys. (DOCX)

S2 Fig Location of the gauging stations used to calibrate and validate the SWAT simulations. (DOCX)

S3 Fig a. Histograms of the selected variables for the sites sampled in the historical (white; 637 sites) and current (grey; 1222 sites) surveys. b. Mean (± standard deviation) values of environmental variables in historical and current periods at sampling sites.
(DOCX)

S4 Fig Environmental variables for the historical period and percentage of change between the historical and current periods. (DOCX)

S1 Table Comparison of the values of the environmental variables between the historical and current periods. (DOCX)

S2 Table Default and calibrated values of snow parameters used to calibration SWAT simulations. (DOCX)
