# Supplementary material for: Can Recent Global Changes Explain the Dramatic Range Contraction of an Endangered Semi-Aquatic Mammal Species in the French Pyrenees?
Source: PLoS One. 2016 Jul 28;11(7):e0159941. doi: 10.1371/journal.pone.0159941 (PMC4965056; doi:10.1371/journal.pone.0159941)
Supplement: S1 Fig — (DOCX) [file pone.0159941.s001.docx]

**S1 Fig.** Observed presence and absence of the Pyrenean desman for the (a) historical (637 sampling sites) and (b) current (1222 sampling sites) periods.
