# Supplementary material for: Can Recent Global Changes Explain the Dramatic Range Contraction of an Endangered Semi-Aquatic Mammal Species in the French Pyrenees?
Source: PLoS One. 2016 Jul 28;11(7):e0159941. doi: 10.1371/journal.pone.0159941 (PMC4965056; doi:10.1371/journal.pone.0159941)

**S2** **Fig.** Location of the gauging stations used to calibrate and validate SWAT simulations. They were selected in order to be evenly spatially distributed over the study area, located generally downstream of the catchment to have large drainage surface and also according to the accuracy of data measured.


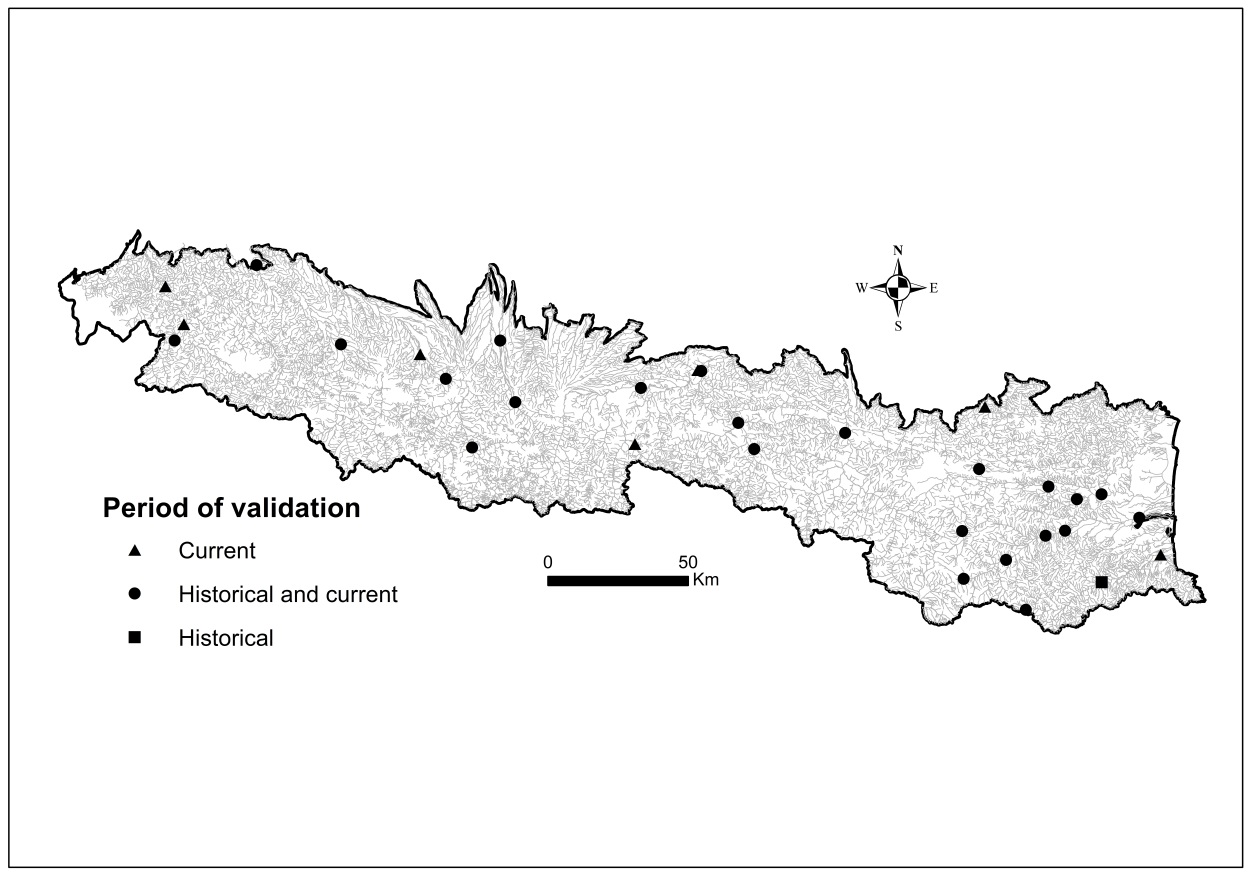

Supplement: S2 Fig — (DOCX) [file pone.0159941.s002.docx]
