# Supplementary material for: Can Recent Global Changes Explain the Dramatic Range Contraction of an Endangered Semi-Aquatic Mammal Species in the French Pyrenees?
Source: PLoS One. 2016 Jul 28;11(7):e0159941. doi: 10.1371/journal.pone.0159941 (PMC4965056; doi:10.1371/journal.pone.0159941)
Supplement: S3 Fig — a. Histograms of the selected variables for the sites sampled in the historical (white; 637 sites) and current (grey; 1222 sites) surveys. b. Mean (± standard deviation) values of environmental variables in historical and current periods at sampling sites. (DOCX) [file pone.0159941.s003.docx]

**S3 Fig.** a. Histograms of the selected variables for the sites sampled in the historical (white; 637 sites) and current (grey; 1222 sites) surveys. (TRI, number of tributaries; URB, proportion of urban areas; AGR, proportion of agricultural lands; FOR, proportion of forested areas; NAT, proportion of open space areas with little or no vegetation; SLO, mean slope of the section; POP, human population density; OBS, density of obstacles to water flow upstream; RAI, mean annual rainfall; TEM, mean annual temperature; FLO, mean monthly flow)

b. Mean (± standard deviation) values of environmental variables in historical and current periods at sampling sites.

| Variable | Historical (mean ± sd) | Current (mean ± sd) |
| --- | --- | --- |
| TEM (°C) | 10.19 (± 2.27) | 10.50 (± 2.55) |
| RAI (mm) | 1296.87 (± 285.44) | 1147.742 (± 241.79) |
| URB (%) | 4.73 (± 13.90) | 3.29 (± 11.68) |
| AGR (%) | 44.21 (± 37.77) | 37.71 (± 37.41) |
| FOR (%) | 37.07 (± 34.17) | 39.97 (± 35.68) |
| NAT (%) | 13.52 (± 26.29) | 18.56 (± 31.63) |
| FLO (m^3^/s) | 6.41 (± 13.29) | 2.45 (± 7.55) |
