# Supplementary material for: Can Recent Global Changes Explain the Dramatic Range Contraction of an Endangered Semi-Aquatic Mammal Species in the French Pyrenees?
Source: PLoS One. 2016 Jul 28;11(7):e0159941. doi: 10.1371/journal.pone.0159941 (PMC4965056; doi:10.1371/journal.pone.0159941)
Supplement: S4 Fig — (DOCX) [file pone.0159941.s004.docx]

**S4 Fig.** Environmental variables for the historical period (left-hand) and percentage of change over the study period (right-hand): (a, b) stream flow, (c, d) temperature, (e, f) rainfall, (g, h) proportion of agricultural lands, (i, j) proportion of forests, (k, l) proportion of urban areas, and (m, n) proportion of open areas. For the maps showing the percentage of change, a negative value indicates that the current value is lower than the historical value whereas a positive value indicates that the current value is higher than the historical value.

|  |
| --- |
|  |
