# Supplementary material for: Can Recent Global Changes Explain the Dramatic Range Contraction of an Endangered Semi-Aquatic Mammal Species in the French Pyrenees?
Source: PLoS One. 2016 Jul 28;11(7):e0159941. doi: 10.1371/journal.pone.0159941 (PMC4965056; doi:10.1371/journal.pone.0159941)
Supplement: S1 Table — (DOCX) [file pone.0159941.s005.docx]

**S1 Table** Environmental variables calculated for the historical and current periods. Mean, standard deviation (SD) and range were computed across the whole study area (i.e. the French Pyrenees). Land-use surface variables were calculated within a 100 m-buffer around each 1 km-long river section. The number of tributaries was calculated for the focal section and the adjacent upstream and downstream sections.

| Variable | Code | Units | Period | Mean | SD | Range | Source |
| --- | --- | --- | --- | --- | --- | --- | --- |
|  |  |  |  |  |  |  |  |
| *Climate* |  |  |  |  |  |  |  |
| Mean annual temperature | TEM | °C | Historical | 10.81 | 2.82 | 1.62-15.37 | CERFACS (Pagé *et al*., 2009) |
|  |  |  | Current | 11.30 | 2.79 | 2.18-15.83 |  |
| Mean annual rainfall | RAI | mm | Historical | 1217.6 | 324.96 | 536.71-2265.06 | CERFACS (Pagé *et al*., 2009) |
|  |  |  | Current | 1049.82 | 277.15 | 508.06-1954.54 |  |
|  |  |  |  |  |  |  |  |
| *Land use* |  |  |  |  |  |  |  |
| Agriculture | AGR | % | Historical | 37.37 | 0.39 | 0-100 | Corine land Cover DB (2006) |
|  |  |  | Current | 37.03 | 0.39 | 0-100 |  |
| Forest | FOR | % | Historical | 38.04 | 0.37 | 0-100 | Corine land Cover DB (2006) |
|  |  |  | Current | 38.13 | 0.37 | 0-100 |  |
| Urban areas | URB | % | Historical | 2.44 | 0.11 | 0-100 | Corine land Cover DB (2006) |
|  |  |  | Current | 2.77 | 0.12 | 0-100 |  |
| Semi-natural areas (from open space to bushy vegetation) | NAT | % | Historical | 21.51 | 0.34 | 0-100 | Corine land Cover DB (2006) |
|  |  |  | Current | 21.32 | 0.34 | 0-100 |  |
|  |  |  |  |  |  |  |  |
| *Hydrology* |  |  |  |  |  |  |  |
| Mean monthly stream flow | FLO | m^3^/s | Historical | 1.69 | 8.38 | 0-140.38 | SWAT simulations |
|  |  |  | Current | 1.47 | 7.20 | 0-116.29 |  |
|  |  |  |  |  |  |  |  |
| *Hydrography* |  |  |  |  |  |  |  |
| Number of tributaries | TRI |  | Static | 1.72 | 1.63 | 0-16 | CARTHAGE ® DB (2011) |
| Mean slope | SLO | % | Static | 12.33 | 14.77 | 0-101.18 | ALTI ® DB (IGN, 2011) |
|  |  |  |  |  |  |  |  |
| *Human disturbance* |  |  |  |  |  |  |  |
| Human population density | POP | number/km² | Static | 54.24 | 151.82 | 0.00-2950.45 | GEOFLA® DB (version 1.1, 2014) |
| Upstream density of obstacles to water flow | OBS | number/km | Static | 0.03 | 0.14 | 0-4.51 | ROE, version 6, 2013 |
