# Supplementary material for: Can Recent Global Changes Explain the Dramatic Range Contraction of an Endangered Semi-Aquatic Mammal Species in the French Pyrenees?
Source: PLoS One. 2016 Jul 28;11(7):e0159941. doi: 10.1371/journal.pone.0159941 (PMC4965056; doi:10.1371/journal.pone.0159941)
Supplement: S2 Table — (DOCX) [file pone.0159941.s006.docx]

**S2** **Table.** Default and calibrated values of snow parameters used for the SWAT simulations.

| **SWAT parameters** | **Description** | **Default values** | **Calibrated values** |
| --- | --- | --- | --- |
| SFTMP | Snow fall temperature | 1.0°C | 1.3°C |
| SMTMP | Snow melt temperature | 0.5°C | 1.97°C |
| SNOCOVMX | Snow water content for 100% snow cover | 1.0 mmH_2_O | 38.38 mmH_2_O |
| SNOW50COV | Fraction of SNOCOVMX corresponding to 50% snow cover | 0.5 | 0.5 |
| SMFMX | Snow melt factor on June 21 | 4.5 mmH_2_O/°C-day | 4.96 mmH_2_O/°C-day |
| SMFMN | Snow melt factor on December 21 | 4.5 mmH_2_O/°C-day | 3.16 mmH_2_O/°C-day |
| TIMP | Snowpack temperature lag factor | 1.0 | 0.14 |
